# Human Mitochondrial Chaperone (mtHSP70) and Cysteine Desulfurase (NFS1) Bind Preferentially to the Disordered Conformation, Whereas Co-chaperone (HSC20) Binds to the Structured Conformation of the Iron-Sulfur Cluster Scaffold Protein (ISCU)*

**DOI:** 10.1074/jbc.M113.482042

**Published:** 2013-08-12

**Authors:** Kai Cai, Ronnie O. Frederick, Jin Hae Kim, Nichole M. Reinen, Marco Tonelli, John L. Markley

**Affiliations:** From the ‡ Center for Eukaryotic Structural Genomics and; § National Magnetic Resonance Facility at Madison, Biochemistry Department, University of Wisconsin-Madison, Madison, Wisconsin 53706

**Keywords:** ATPases, Chaperone Chaperonin, Enzyme Catalysis, Mitochondria, NMR, Protein Conformation, Protein-Protein Interactions, Scaffold Proteins, Spectroscopy

## Abstract

Human ISCU is the scaffold protein for mitochondrial iron-sulfur (Fe-S) cluster biogenesis and transfer. NMR spectra have revealed that ISCU populates two conformational states; that is, a more structured state (S) and a partially disordered state (D). We identified two single amino acid substitutions (D39V and N90A) that stabilize the S-state and two (D39A and H105A) that stabilize the D-state. We isolated the two constituent proteins of the human cysteine desulfurase complex (NFS1 and ISD11) separately and used NMR spectroscopy to investigate their interaction with ISCU. We found that ISD11 does not interact directly with ISCU. By contrast, NFS1 binds preferentially to the D-state of ISCU as does the NFS1-ISD11 complex. An *in vitro* Fe-S cluster assembly assay showed that [2Fe-2S] and [4Fe-4S] clusters are assembled on ISCU when catalyzed by NFS1 alone and at a higher rate when catalyzed by the NFS1-ISD11 complex. The DnaK-type chaperone (mtHSP70) and DnaJ-type co-chaperone (HSC20) are involved in the transfer of clusters bound to ISCU to acceptor proteins in an ATP-dependent reaction. We found that the ATPase activity of mtHSP70 is accelerated by HSC20 and further accelerated by HSC20 plus ISCU. NMR studies have shown that mtHSP70 binds preferentially to the D-state of ISCU and that HSC20 binds preferentially to the S-state of ISCU.

## Teaser

**Background:** Iron-sulfur cluster biosynthesis involves a scaffold protein (ISCU), cysteine desulfurase (NFS1), chaperone (mtHSP70), and co-chaperone (HSC20).

**Results:** Human mitochondrial ISCU populates structured (S) and disordered (D) conformational states. S interacts preferentially with NFS1 and mtHSP70; D interacts preferentially with HSC20.

**Conclusion:** Shifts in the S ⇄ D equilibrium reveal functional states.

**Significance:** The scaffold protein metamorphic property seen in *Escherichia coli* is conserved in humans.

## Introduction

Fe-S clusters are ancient protein prosthetic groups that participate in a wide variety of biological processes, including electron transfer, substrate binding and activation, redox catalysis, DNA replication and repair, regulation of gene expression, and tRNA modification (1–3). In bacteria, three Fe-S cluster biogenetic systems have been discovered: the NIF (nitrogen fixation) system involved in maturation of nitrogenase, the SUF (sulfur mobilization) system encoded by the *suf* operon and highly active under oxidative stress conditions, and the ISC^2^ (iron sulfur cluster) system, the general Fe-S cluster biosynthetic pathway (for review, see Refs. 4, 5, and 7–10). Among the three systems, the ISC system is the best-studied and is believed to be the “housekeeping” biosynthetic system (5). The *isc* operon of *Escherichia coli* codes for several proteins: a repressor (IscR), a cysteine desulfurase (IscS), a scaffold protein (IscU), a protein proposed to be an alternative scaffold (IscA), a DnaJ-type co-chaperone (HscB), a DnaK-type chaperone (HscA), a ferredoxin (Fdx), and a protein of uncertain function (IscX). By catalyzing the conversion of l-cysteine to l-alanine, the pyridoxal-5′-phosphate-dependent enzyme IscS generates S^0^, which is transferred to Cys-328 to form a persulfide and then transferred to IscU (11, 12). Iron is added to form a [2Fe-2S] cluster. IscU-[2Fe-2S] then binds to HscB, which targets it to the HscA-ATP complex. In a reaction involving hydrolysis of ATP, the cluster is transferred to an acceptor protein such as apoferredoxin (13, 14). We have shown by NMR studies that *E. coli* IscU populates two interconvertible conformational states: a more structured state (S) and a partially disordered state (D) (15). The two states play different roles in the cycle of Fe-S cluster assembly and transfer. The D-state is the substrate for IscS (16); the S-state is the form that binds a [2Fe-2S] cluster (17) and binds preferentially to HscB (15, 18). Upon hydrolysis of ATP, HscA binds to the D-state of IscU, ensuing complete release of the cluster to an acceptor protein.

**FIGURE 1.**
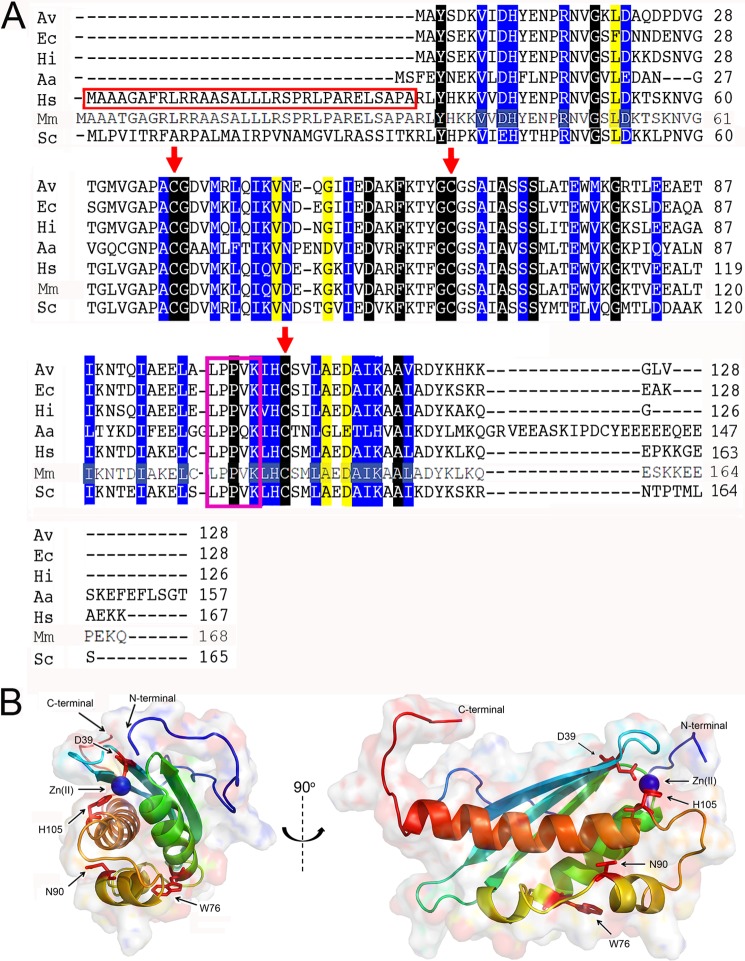
**Alignment of sequences of IscU homologues and structural representation of ISCU.** *A*, alignment of sequences of IscU homologues. Analysis (15) of a much larger set of aligned sequences than those represented here showed that the residues highlighted in *black* were identically conserved, those in *blue* were conserved, and those in *yellow* were semi-conserved. The conserved cysteine residues are marked with *red arrows*, and the conserved LPPVK motif recognized by chaperone proteins is *boxed in magenta*. Mitochondrial proteins contain an N-terminal sequence that targets ISCU to cross the inner mitochondrial membrane. Excluding this region (*boxed in red*), human ISCU and *E. coli* IscU share 77% sequence identity. The numbering systems for mature ISCU and *E. coli* IscU are identical. Abbreviations used are: *Av*, *A. vinelandii; Ec*, *Escherichia coli*; *Hi*, *H. influenzae*; *Aa*, *A. aeolicus*; *Hs*, *Homo sapiens*; *Mm*, *M. musculus*; *Sc*, *S. cerevisiae. B*, solution structure of Zn^2+^ bound *M. musculus* ISCU (PDB code 1WFZ) (47), which shares ∼98% sequence identity with human ISCU, provides insights into the structure of human ISCU. Similar to other IscU homologues, *M. musculus* ISCU consists of three β-strands and four α-helices. Residues mutated in this study (Asp-39, Asn-90, and His-105) are shown in *red stick format*. Asp-39 and His-105 are close to the Zn^2+^ binding site, and Asn-90 is a solvent-exposed polar residue located in a hydrophobic region. All three residues are highly conserved among IscU homologues, and amino acid substitutions at these sites in *E. coli* IscU have been shown to perturb the position of the D ⇄ S conformational equilibrium (35). Another important residue Trp-76 is shown in *red stick* format.

ISCU-type proteins are highly conserved throughout living systems (Fig. 1*A*). Mitochondria contain an ISC-type Fe-S cluster assembly and transfer system, parts of which are homologous to the ISC system of prokaryotes (6). In humans it has been proposed that this system involves a scaffold protein (ISCU) (19), a cysteine desulfurase consisting of two subunits, NFS1 (homologous to IscS) and ISD11 (no bacterial homologue found), a DnaJ-type co-chaperone (HSC20), and a DnaK-like chaperone (mtHSP70) (6, 19). These mitochondrial proteins are synthesized in the cytoplasm with N-terminal extensions that facilitate mitochondrial entry and are cleaved off upon protein maturation in mitochondria (20). The mature form of human ISCU, which shares 77% sequence identity with *E. coli* IscU (Fig. 1*A*), has been shown to play an important role in cellular iron homeostasis (21). A tissue-specified splicing mutation of human ISCU has been associated with the disease ISCU myopathy (22). It has been proposed that the small protein ISD11 stabilizes the cysteine desulfurase NFS1 (23) and is important for Fe-S cluster assembly and cellular iron homeostasis (24, 25). Although it has been shown in an *in vitro* assay that the ISCU-NFS1-ISD11 complex can assemble Fe-S clusters (26), little is known about the interactions among these three proteins. Human mtHSP70 (also termed Mortalin, PBP74, GRP75, or HSPA9) appears to be a multifunctional DnaK-type chaperone (27) (unlike *E. coli* HscA, which is involved only in Fe-S cluster biogenesis). Human mtHSP70 is known to perform other cellular functions, including protein folding, intracellular trafficking, antigen processing, and aging (28, 29). It has been shown that mtHSP70 binds a variety of substrates, including cancer suppressor protein p53 and Parkinson disease-related protein DJ-1, and mtHSP70 has been associated with Alzheimer disease (30, 31). Because mtHSP70 is the only Hsp70 chaperone present in human mitochondria, it has been suggested that it plays a role in the human ISC machinery similar to that of *E. coli* HscA (32). This hypothesis is supported by the finding that the mtHSP70 homolog in *Saccharomyces cerevisiae* (Ssc1) participates in Fe-S cluster biosynthesis when the specialized Hsp70 chaperone (Ssq1) is knocked out (33). HSC20 recently has also been found to be involved in human mitochondrial Fe-S cluster biogenesis (34).

Given the sequence similarities of the bacterial and human proteins, it was of interest to determine, whether the human scaffold protein (ISCU) shares the conformational duality of the *E. coli* ortholog (IscU) and, if so, whether the two states exhibit differential interaction with the cysteine desulfurase, co-chaperone, and chaperone proteins. We report here that the human scaffold protein for Fe-S cluster biogenesis (ISCU), like its *E. coli* counterpart (IscU), populates two conformational states, a more structured state (S) and a partially disordered state (D). However, the relative population of the S-state of human ISCU is lower (28%) than the corresponding population of *E. coli* IscU (80%) under comparable solution conditions. Human cysteine desulfurase (NFS1) alone and in the NFS1-ISD11 complex were found to bind preferentially to the D-state of ISCU. However, ISCU does not interact directly with ISD11. The co-chaperone (HSC20) was found to bind preferentially to the S-state of ISCU, whereas the chaperone (mtHSP70) was found to bind preferentially to the D-state of ISCU. HSC20 activated the ATPase activity of mtHSP70, and this activation was greatly increased by the addition of ISCU.

## EXPERIMENTAL PROCEDURES

### 

#### 

##### DNA Cloning

The cDNAs encoding the human mitochondrial proteins ISCU, NFS1, ISD11, mtHSP70, and HSC20 were ordered from the Mammalian Gene Collection (Thermo Fisher; Pittsburgh, PA). The genes coding for bacterial proteins were isolated by PCR directly from *E. coli* genomic DNA (Sigma). The clones for the mitochondrial proteins were designed to yield the mature protein sequences; the gene sequence coding for N-terminal mitochondrial targeting peptide was excluded (Fig. 1*A, red box*), and the clones coded for a SUMO fusion containing an N-terminal His-tag. The genes were cloned into the pE-SUMO-Kan vector (Lifesensors; Malvern, PA) by using BsaI and XhoI restriction sites in the PCR gene fragments and vector. DNA primers used in these experiments were ordered through either the University of Wisconsin-Madison Biotechnology Center or Integrated DNA Technologies, Inc. (Coralville, IA). Restriction enzymes were purchased from either Promega (Madison, WI) or New England Biolabs (Ipswich, MA). All PCR DNA primers used for cloning included nine additional base pairs at the 5′ ends upstream of the restriction sites to make digestion more efficient at the termini of PCR products. DNA ligation and construction of the expression plasmid were carried out in 10-μl reactions with a PCR-based ligation thermo cycling program (30 s at 30 °C and 30 s at 10 °C repeated 800 times for 12 h). The ligation reaction was heat-inactivated at 65 °C for 25 min and then used to transform chemically competent 10G cells (Lucigen; Madison, WI), which were plated onto YT plates (containing 50 μg/ml kanamycin) and incubated at 37 °C overnight. An *E. coli* recombinant colony archive was constructed by picking individual colonies and storing them in 30 μl of 20% sterile glycerol. 3-μl aliquots were removed for use in a PCR colony screen (20-μl reaction using Promega 2× PCR master mix) to identify positive clones. The original PCR primers used for isolating the target genes were employed in this PCR colony screen, and the reactions were analyzed on 1% agarose gels in TAE buffer (40 mm Tris acetate, 1 mm EDTA, pH 8.0). To prepare plasmids for DNA sequence analysis, the *E. coli* colony glycerol stocks that yielded positive recombinants according to the PCR colony screens were grown overnight at 37 °C in 2–3 ml of CircleGrow® broth (MP Biomedicals; Santa Ana, CA) in the presence of 50 μg/ml kanamycin. All DNA sequencing reactions were carried out on a Bio-Rad Dyad Peltier Thermal cycler at the University of Wisconsin-Madison Biotechnology Center, and SeqMan software (DNASTAR; Madison, WI) was used to analyze and identify targets with the correct DNA sequence.

We chose sites for production of single amino acid variants of ISCU to match those known to affect the D ⇄ S conformational equilibrium of *E. coli* IscU (35). The positions of these residues on *Mus musculus* Zn^2+^ bound ISCU (a model for the S-state) are shown in Fig. 1*B* (PDB code 1WFZ). *M. musculus* ISCU shares 98% sequence identity with human ISCU (Fig. 1*A*). Genes for these variants were produced by using the Polymerase Incomplete Primer Extension (PIPE) site-directed mutagenesis method (36).

##### Production of Proteins

Single colonies containing validated genes for the target proteins (human ISCU variants, NFS1, ISD11, mtHSP70, and HSC20 and *E. coli* IscS) were picked from the YT or MDAG plates (37) and grown in 1 ml of CircleGrow® broth or YT with 1% glucose (plus appropriate antibiotics) for 1–3 h at 37 °C at 250 rpm and then transferred to 50–100 ml of MDAG medium (with appropriate antibiotics) and grown overnight at 25 °C. For large scale protein production, 1 liter of unlabeled Terrific Broth (TB) auto-inducing medium or M9 isotopic medium was prepared, and 500-ml aliquots were transferred into sterile PET soda bottles (38, 39). Each 500-ml aliquot was inoculated with 10 ml of the overnight MDAG starter culture, and the cell cultures were grown at 37 °C (250–320 rpm) for 2–5 h before dropping the growth temperature to 10–25 °C for 24–36 h. For induction with isopropyl-1-thio-β-d-galactoside (IPTG), the cell cultures were grown to *A*_600_ of 1.0–1.5; then the temperature was dropped to 10–25 °C, and IPTG (0.1–0.2 mm final concentration) was added when the temperature had stabilized (after about 15–30 min). For stable isotope labeling, we used an M9-based medium consisting of 100 ml/liter 10× M9 salts (70 g/liter Na_2_HPO_4_, 30 g/liter KH_2_PO_4_, and 5 g/liter NaCl), 1 ml of 1000× metal mix, 1 ml of vitamin mixture (40), 30 mg/liter thiamine, 0.5 ml of 0.2 m CaCl_2_ (0.1 mm final concentration), 2–5 drops of sterile antifoam, 2 ml of 1 m MgSO_4_ (2 mm final concentration), [U-^13^C]glucose (2–4 g/liter), ^15^NH_4_Cl (1 g/liter), plus the appropriate antibiotics (chloramphenicol to 35 μg/ml and kanamycin to 50–100 μg/ml). At the end of cell growth, the cultures were harvested by centrifugation for 30 min at 4000 × *g* in a centrifuge with a JS-4.0 rotor (Beckman Coulter; Brea, CA). The cell pastes were stored at −80 °C until needed for protein purification.

##### Buffers

The composition of the 1st immobilized metal affinity chromatography (IMAC) buffer was 20 mm Tris-HCl, pH 8, 300–500 mm NaCl, 0.1% Nonidet P-40, 1–2 mm β-mercaptoethanol or DTT, 1 mm phenylmethanesulfonyl fluoride (PMSF), 5–10% glycerol, and 5 mm imidazole. The composition of the 2nd IMAC buffer was the same as the 1st IMAC buffer except that it contained 250 mm imidazole. The SUMO-fusion cleavage buffer contained 20 mm Tris buffer at pH 8, 150 mm NaCl, 2 mm DTT (or β-mercaptoethanol), and 5–10% glycerol. The TND buffer consisted of 50 mm Tris-HCl, pH 8, containing 150 mm NaCl, 5 mm DTT, and 0.3% NaN_3_. The TKDM buffer consisted of 50 mm Tris-HCl, pH 7.5, containing 150 mm KCl, 5 mm DTT, and 10 mm MgCl_2_.

##### Protein Purification

All *E. coli* cell pastes were quickly thawed either on ice or at room temperature and then resuspended in 60–70 ml of lysis buffer: 1st IMAC buffer supplemented with Benzonase (Novagen, Millipore; Billerica, MA) or OmniCleave nuclease (Epicenter, Illumina; Madison, WI), rLysozyme (Novagen), RNase (Qiagen; Valencia, CA), and 0.1% Nonidet P-40 (Sigma). To break open the resuspended cells, we used sonication with a total time of 15–30 min at 4 °C, with a duty cycle of 2 s on and 4 s off. Cell lysates were clarified by high-speed centrifugation at 25,000 rpm for 30 min in a centrifuge with a JA 30.5Ti rotor (Beckman Coulter). The clear cell lysate was then treated with 0.1% w/v polyethylene imine (PEI) to precipitate RNA and then was spun again for 30 min at 25,000 rpm and eluted. DTT was added to a level of 2 mm to ensure reduction of cysteines. The cell lysate was refrigerated at 4 °C, and (NH_4_)_2_SO_4_ was added to 70% (w/v) saturation to precipitate total protein and to remove PEI. Then the sample was spun at 25,000 rpm for 30 min. The protein pellet was resuspended in 30–50 ml of 1st IMAC buffer (without NaCl but with 2 mm DTT), and any debris was discarded after a final centrifugation at 25,000 rpm for 30 min. The clarified protein solution was loaded onto a Qiagen Superflow FF or Ni-Sepharose column (GE Healthcare) IMAC resin at 1–5 ml/min. The IMAC column was washed first with ∼10 column volumes of 1st IMAC buffer and second with 5–10 column volumes of wash buffer (1st IMAC buffer + 30 mm imidazole). The target protein was eluted with the 2nd IMAC buffer, and fractions were collected. SDS-PAGE was used to analyze and assess the purity of the eluted target protein in the collected fractions.

The His-tagged, N-terminal SUMO fusion protein was digested with 0.5 mg of SUMO protease. The reaction was carried out in SUMO-fusion cleavage buffer either by desalting the fusion protein by size exclusion chromatography and adding SUMO protease or, more usually, by dialyzing the fusion protein in the presence of SUMO protease overnight at 4 °C against the cleavage buffer. The cleaved sample was loaded onto a freshly equilibrated subtractive IMAC column, which bound the cleaved His-tagged SUMO domain and allowed the cleaved target protein to pass through to a fraction collector. The purities of the target protein fractions were assessed by SDS-PAGE. Mass spectrometry was used to determined target protein masses and the level of stable isotope incorporation.

##### NMR Spectroscopy

For NMR samples the TND and TKDM buffers were modified to contain 10% D_2_O for the frequency lock. All NMR spectra were collected on 600 MHz (^1^H) Bruker BioSpin (Billerica, MA) NMR spectrometers equipped with a *z*-gradient cryogenic probe. All sample temperatures were regulated at 25 °C. NMRPipe software (41) was used to process the raw NMR data, and SPARKY software (42, 43) was utilized to visualize and analyze the processed NMR data.

^1^H,^15^N HSQC spectra of wild-type (WT) and variant ISCU samples were collected with 0.3 mm U-^15^N-ISCU in TND buffer. To monitor the effects of added unlabeled NFS1 or ISD11, we first collected a ^1^H,^15^N HSQC spectrum 0.5 mm U-^15^N-ISCU in TND buffer. Then an equal volume of 0.5 mm unlabeled NFS1 was first added, and a ^1^H,^15^N TROSY-HSQC spectrum was acquired. Next, an equal volume of 0.5 mm unlabeled ISD11 in TND buffer was added, and another ^1^H,^15^N TROSY-HSQC spectrum was acquired. Because of the dilution effect, the intensities of the ^1^H,^15^N peaks from ISCU diminished by a factor of two after the addition of NFS1 and by a factor of 3 after the addition of ISD11. However, the quantity of interest was the effect on the relative population of the S-state.

The titrations with mtHSP70 were started by collecting ^1^H,^15^N HSQC spectra of samples of 0.5 mm U-^15^N-ISCU and 0.5 mm U-^15^N-ISCU(N90A) in TKDM buffer. Then aliquots of unlabeled 0.5 mm mtHSP70 in TKDM buffer were added to each sample, and ^1^H,^15^N TROSY-HSQC spectra were acquired. Because of the dilution effect, the intensities of the ^1^H,^15^N peaks from ISCU diminished by a factor of two at equimolar ISCU:mtHSP70. Again, the quantity of interest was the effect on the relative population of the S-state.

The titrations with HSC20 were started by collecting ^1^H,^15^N TROSY-HSQC spectra of samples of 0.5 mm U-^15^N-ISCU or U-^15^N-ISCU(N90A) in TKDM buffer. ^1^H,^15^N TROSY-HSQC spectra were acquired after the addition of aliquots of 0.4 mm unlabeled HSC20 in TKDM buffer. Because of the dilution effect, the intensities of the ^1^H,^15^N peaks diminished by a factor of 2.25 at equimolar ISCU:HSC20. However, the quantity of interest was the ratio [S]/([S] + [D]).

##### Resonance Assignment of ISCU(N90A)

We were able to assign NMR signals of ISCU(N90A), a variant that fully populates the S-state. We collected three-dimensional CBCACONH and HNCACB spectra from a sample of ISCU(N90A) labeled uniformly with ^13^C and ^15^N and used the data to carry out sequential backbone assignments.

##### Determination of the Position of the S ⇄ D Equilibrium

We used the Newton software package (44) to calculate the relative intensities of the Trp-76 cross-peaks from the S- and D-states. Newton carries out fast maximum likelihood reconstruction (FMLR) of two-dimensional NMR signals to provide rigorous signal intensities by fitting their position, amplitude, line width, and phase. The percent of ISCU in the S-state, %S, is given by Equation 1, where [S] is obtained from the intensity of the S peak and [D] is from the intensity of the D peak from Trp-76.

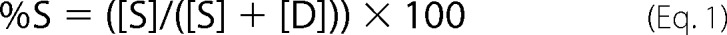
 We carried out three or more independent FMLR analyses of each spectrum to determine reproducibility and estimate errors (shown as error bars).

##### Circular Dichroism Spectroscopy

The sample buffer used in circular dichroism (CD) experiments contained 20 mm NaH_2_PO_4_ and 50 mm NaCl at pH 8. The solutions were placed in 1-mm path length quartz cuvettes. The concentration of ISCU variants was 20 μm. Far-UV CD spectra of ISCU variants were collected with an Aviv 202SF CD spectrophotometer (Aviv Biomedical; Lakewood, NJ) at 25 °C. Secondary structure content was estimated from the CD spectra by using K2D2 software (45).

##### Size Exclusion Chromatography (Gel Filtration)

Analytical gel-filtration studies were conducted with Hi-Load 16/60 Superdex 75 Column (GE Healthcare) at room temperature. To investigate the interaction between HSC20 and ISCU (WT or N90A), a 2:1 (molar ratio) mixture of HSC20:ISCU in TKDM buffer was injected. The protein sample was eluted at 1 ml/min flow rate with TKDM buffer as the elution buffer, and 2 ml fractions were collected using an automatic fraction collector (GE Healthcare). Eluted fractions were analyzed by SDS-PAGE.

##### ATPase Assays

ATPase assays were carried out in TKDM buffer containing 0.1 mm ATP. The ATPase activity of mtHSP70 was determined at 25 °C by using an EnzCheck Phosphate Assay kit (Invitrogen) to measure the rate of phosphate release rate as described previously (46).

##### In Vitro Fe-S Custer Assembly

A published protocol (47) was used to assemble Fe-S clusters *in vitro*. All samples were prepared in an anaerobic chamber (Coy Laboratory; Farmingdale, NY) filled with 90% N_2_ gas and 10% H_2_ gas. The reconstitution mix in TND buffer at pH 7.5 consisted of 50 μm ISCU or ISCU(N90A), 250 μm Fe_2_(NH_4_)_2_(SO_4_)_2,_ and 1 μm cysteine desulfurase (NFS1, NFS1-ISD11, or *E. coli* IscS). The reaction was initiated by adding 250 μm l-cysteine into the reconstitution mix to make the final volume equal 1 ml. The reaction was carried out at 25 °C in a 10-mm path length quartz cuvette sealed with a rubber septum. Spectra were collected on a UV-1700 UV-visible spectrophotometer (Shimadzu; Kyoto, Japan) equipped with a temperature control utility. UVProbe 2.21 software (Shimadzu) was used to collect and analyze the data.

## RESULTS

### 

#### 

##### ISCU Populates Two Conformational States

**FIGURE 2.**
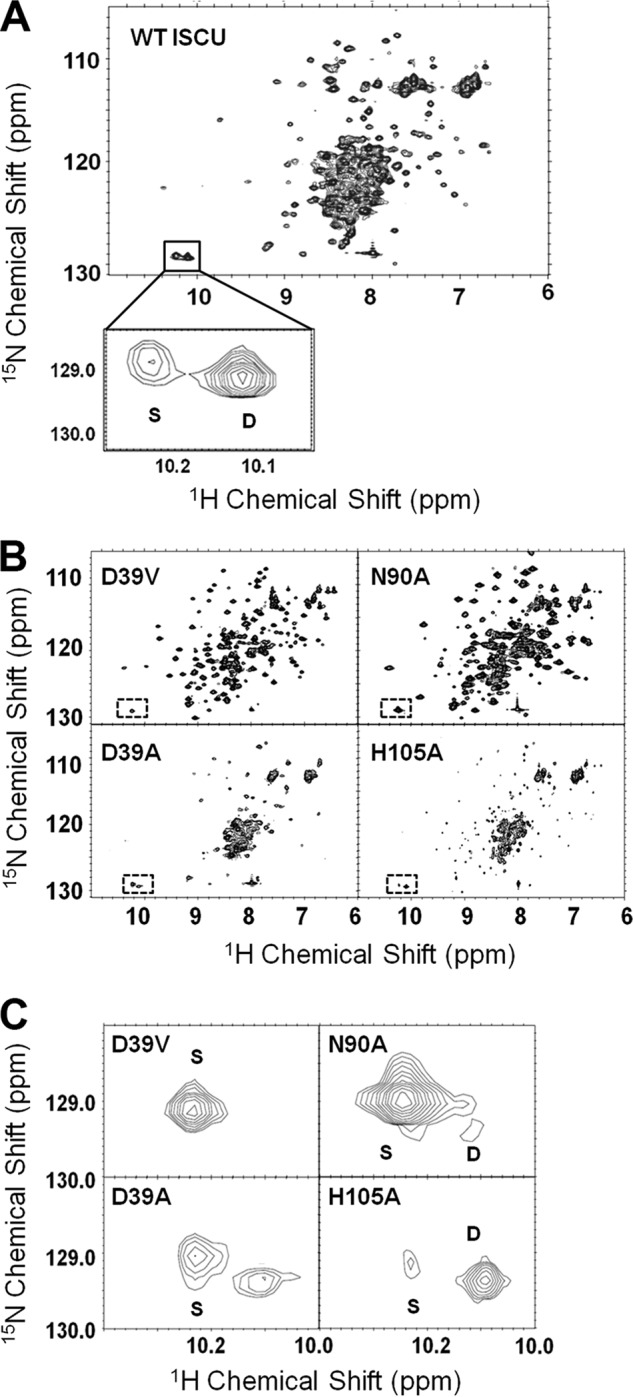
**NMR evidence that ISCU exists in solution as two slow interchanging conformational states and that the S ⇄ D equilibrium is perturbed by single amino acid substitutions.** *A*, ^1^H,^15^N HSQC NMR spectrum of ISCU. Because ISCU contains only one tryptophan (Trp-76), the presence of two cross-peaks in the *boxed region* and *inset* indicates the existence of two different conformational states. Assignments of individual peaks to the S- and D-states are indicated. *B*, ^1^H,^15^N HSQC NMR spectra of ISCU variants with shifted S ⇄ D equilibria. Whereas the substitutions D39V and N90A stabilize the S-state, substitutions D39A and H105A stabilize the D-state. *C*, expansions of the spectra in B show the Trp-76 side chain signals used to quantify the relative populations of the S- and D-states. All NMR spectra were collected at 600 MHz (^1^H) at 25 °C with solutions at pH 8.0.

Evidence for the structural heterogeneity of ISCU came from ^1^H,^15^N HSQC NMR spectra that exhibited two sets of peaks for certain residues. The most prominent of these was the doubled ^1^H,^15^N cross-peak from the side chain of Trp-76, the only tryptophan residue in the protein (*boxed signals* on Fig. 2*A*). The spectral analysis was clarified by comparison of ^1^H,^15^N HSQC spectra of ISCU (Fig. 2*A*) with those of the four single-site mutants (Fig. 2*B*). Variants ISCU(D39V) and ISCU(N90A) yielded ^1^H,^15^N HSQC spectra with sharp, well dispersed peaks, as expected for a well structured protein, whereas variants ISCU(D39A) and ISCU(H105A) yielded ^1^H,^15^N HSQC spectra with broader, poorly dispersed peaks (particularly in the ^1^H dimension), as expected for a partially disordered protein. The spectrum of wild-type ISCU exhibited both sets of peaks. We thus assigned the sharper set of peaks to the structured state (*S*) and the broader set of peaks to the partially disordered state (*D*). Comparison of the Trp-76 peaks from ISCU (Fig. 2*A*) with those from the variants (Fig. 2*C*) allowed us to assign signals to the individual states. The %S values for the ISCU variants studied here are collected in Table 1.

**TABLE 1:** **Properties of wild-type (WT) human ISCU and single-site amino acid variants at pH 8 and 25 °C**

Structural property	WT	D39V	N90A	D39A	H105A
%S (determined by NMR) = ([S]/([S] + [D])) × 100 (%)	27.5	100	95.4	29.1	11.2
Molar ellipticity at 220 nm (10^4^ deg cm^2^ dmol^−1^)	0.742	0.926	1.01	0.568	0.476
Predicted α-helix (%)	16.2	25.5	25.0		
Predicted β-strand (%)	18.2	19.2	20.2		

**FIGURE 3.**
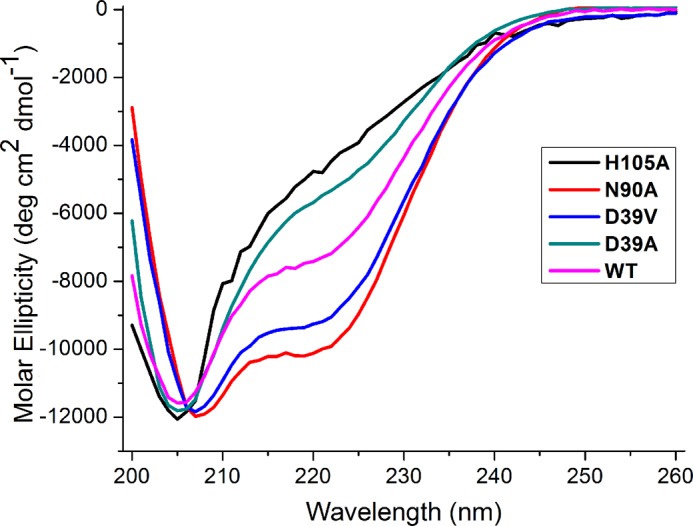
**Far-UV CD spectra of ISCU variants.** All spectra were collected at 25 °C with solutions at pH 8. The CD spectra of ISCU(N90A) (*red*) and ISCU(D39V) (*blue*) indicate the presence of secondary structure, but the CD spectra of ISCU(H105A) (*black*) and ISCU(D39A) (*green*) suggest that little secondary structure is present. The CD spectrum of wild-type ISCU (*magenta*) is intermediate between those of the variants stabilizing the S- and D-states.

We used CD spectroscopy to investigate the secondary structure of the ISCU variants. Far-UV (200–260 nm) CD spectra of ISCU variants that stabilize the S-state as shown by NMR exhibited secondary structure, whereas variants that stabilize the D-state as shown by NMR yielded CD spectra that could not be interpreted in terms of secondary structure (Fig. 3; Table 1). The CD spectrum of wild-type ISCU was consistent with the mixed population determined by NMR of ∼28% S-state and 72% D-state. The CD spectra of the structured variants of human ISCU (D39V and N90A) as analyzed by K2D2 software (43) yielded ∼25% α-helix and ∼20% β-strand (Table 1). By comparison, the solution structure of Zn^2+^-bound *M. musculus* IscU (PDB code 1WFZ, 10.2210/pdb1wfz/pdb), a model for the S-state, contained ∼41% α-helix and ∼20% β-strand.

##### Interaction of ISCU with NFS1 and ISD11

**FIGURE 4.**
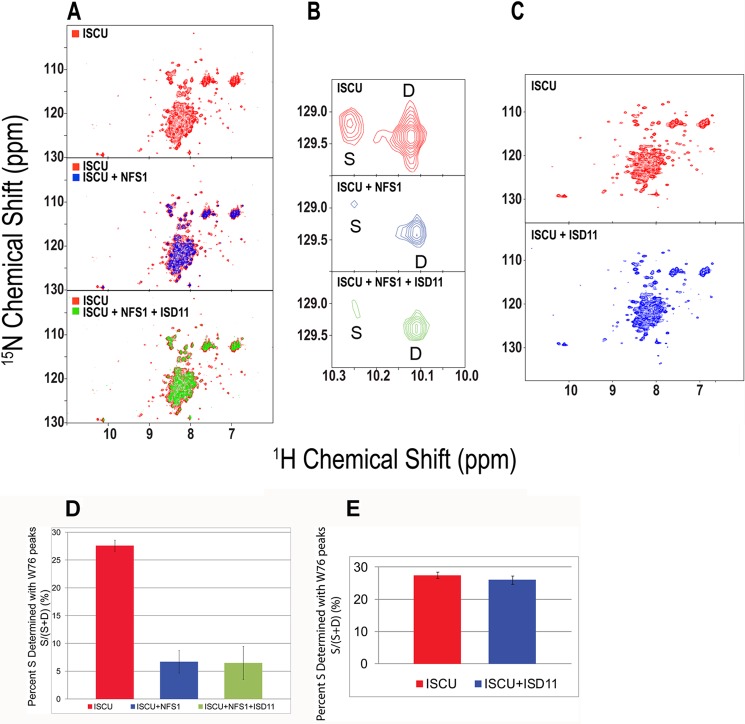
**Evidence that NFS1 binds preferentially to the D-state of ISCU.** *A*, *top panel*, two-dimensional ^1^H,^15^N HSQC spectrum of U-^15^N-ISCU. *Middle panel*, overlay of the two-dimensional ^1^H,^15^N TROSY-HSQC spectra of U-^15^N-ISCU alone (*red*) and U-^15^N-ISCU (*blue*) mixed with a stoichiometric amount of unlabeled NFS1. *Bottom panel*, overlay of two-dimensional ^1^H,^15^N TROSY-HSQC spectra of U-^15^N-ISCU (*red*) and the same sample mixed with stoichiometric amounts of unlabeled NFS1 and ISD11 (*green*). *B*, expansions show the ^1^H,^15^N peaks from the indole ring of Trp-76 of ISCU. *Top panel*, U-^15^N-ISCU. *Middle panel*, U-^15^N-ISCU mixed with equimolar unlabeled NFS1. *Bottom panel*, U-^15^N-ISCU mixed with equimolar unlabeled NFS1 and ISD11. *C*, *top panel*, two-dimensional ^1^H,^15^N HSQC spectrum of U-^15^N-ISCU. *Bottom panel*, two-dimensional ^1^H,^15^N HSQC spectrum of U-^15^N-ISCU mixed with equimolar unlabeled ISD11. All NMR spectra were collected at 600 MHz (^1^H) at 25 °C with samples at pH 8.0. *D* and *E*, %S calculated by FMLR analysis of the intensities of Trp-76 cross-peaks assigned to the S- and D-states under the conditions indicated.

Two-dimensional ^15^N TROSY-HSQC NMR spectra of U-^15^N-ISCU were collected before and after adding a stoichiometric amount of unlabeled NFS1. The %S determined by FMLR analysis of the relative intensities of the Trp-76 peaks assigned to the S- and D-states (Fig. 4, *A* and *B*) decreased from ∼27 to ∼7% upon adding 1 eq of NFS1 and remained unchanged after the subsequent addition of 1 eq of ISD11 (Fig. 4*D*). This result indicates that NFS1 binds preferentially to the D-state of ISCU. The addition of unlabeled ISD11 to U-^15^N-ISCU led to no changes in the two-dimensional ^15^N TROSY-HSQC NMR spectra (Fig. 4, *C* and *E*), which indicated that ISCU and ISD11 do not interact directly.

##### mtHSP70 Binds Preferentially to the D-state of ISCU

**FIGURE 5.**
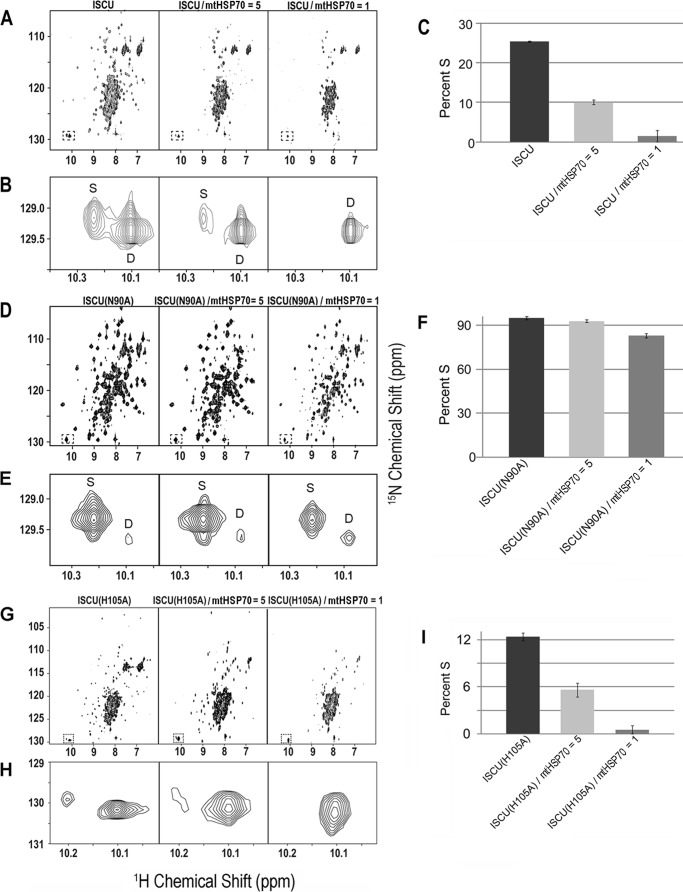
**Evidence that mtHSP70 binds preferentially to the D-state of ISCU.** *A*, two-dimensional ^1^H,^15^N HSQC spectra of U-^15^N-ISCU (*left panel*), U-^15^N-ISCU sample mixed with 0.2 eq of unlabeled mtHSP70 and diluted by a factor of 1.2 (*middle panel*), U-^15^N-ISCU mixed with 1.0 eq of mtHSP70 and diluted by a factor of 2 (*right panel*). *B*, expansions of the Trp-76 ^1^H,^15^N cross-peaks from the spectra in *A. C*, %S calculated by FMLR analysis of the intensities of the Trp-76 cross-peaks assigned to S and D under the conditions indicated. *D*, two-dimensional ^1^H,^15^N TROSY-HSQC spectra of U-^15^N-ISCU(N90A) (*left panel*), U-^15^N-ISCU(N90A) plus 0.2 eq of unlabeled mtHSP70 and diluted by a factor of 1.2 (*middle panel*), U-^15^N-ISCU(N90A) mixed with 1.0 eq of mtHSP70 and diluted by a factor of 2 (*right panel*). *E*, expansions of the Trp-76 ^1^H,^15^N cross-peaks from the spectra in *D. F*, %S calculated by FMLR analysis (44) of the intensities of the Trp-76 cross-peaks assigned S and D under the conditions indicated. *G*, two-dimensional ^1^H,^15^N TROSY-HSQC spectra of U-^15^N-ISCU(H105A) (*left panel*), U-^15^N-ISCU(H105A) mixed with 0.2 eq of unlabeled mtHSP70 and diluted by a factor of 1.2 (*middle panel*), U-^15^N-ISCU(H105A) plus 1.0 eq of unlabeled mtHSP70 and diluted by a factor of 2 (*right panel*). *H*, expansions of the Trp-76 ^1^H,^15^N cross-peaks from the spectra in *G. I*, %S calculated by FMLR analysis of the intensities of the Trp-76 cross-peaks assigned S and D under the conditions indicated. All NMR spectra were collected at 600 MHz (^1^H) at 25 °C with samples at pH 8.0.

**FIGURE 6.**
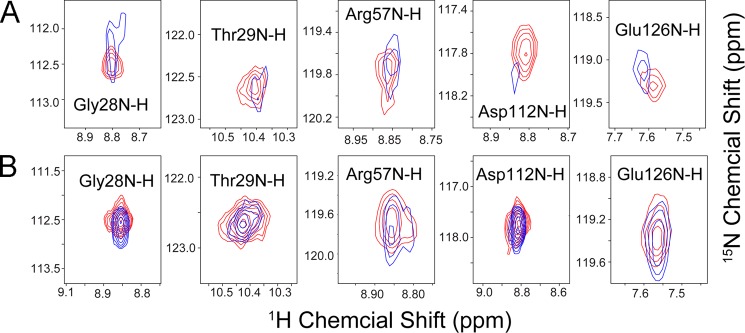
**Effect of added mtHSP70 on selected peaks from ^1^H,^15^N TROSY-HSQC spectra of U-^15^N-ISCU and U-^15^N-ISCU(N90A).** *A*, U-^15^N-ISCU alone (*red*) and U-^15^N-ISCU with 0.2 eq of added unlabeled mtHSP70 (*blue*). *B*, U-^15^N-ISCU(N90A) alone (*red*) and U-^15^N-ISCU(N90A) with 0.2 eq of added unlabeled mtHSP70 (*blue*).

To determine whether mtHSP70, the only Hsp70 chaperone protein in mitochondria (27), interacts with ISCU, we used two-dimensional ^15^N TROSY-HSQC NMR spectroscopy to follow the titration of U-^15^N-ISCU with unlabeled mtHSP70. The addition of mtHSP70 led to a progressive decrease in %S by FMLR analysis (Fig. 5*C* and Table 2). %S decreased from 27% in the absence of mtHSP70 to 1.2% at equimolar ISCU:mtHSP70 (Fig. 5, *A* and *B*). The results indicate that mtHSP70 binds preferentially to the D-state of ISCU. Several ^1^H,^15^N peaks from ISCU were found to be perturbed in the presence of 0.2 eq of mtHSP70 (Fig. 6*A*).

**TABLE 2:** **Effect of added mtHSP70 on the %S of ISCU variants** Values in the table represent %S (determined by NMR) = ([S]/([S] + [D])) × 100 (%).

ICSU variant	Mole fraction of added mtHSP70	
	0	1.0
	%*S*	
ISCU	27.5	1.2
ISCU(N90A)	95.4	83.0
ISCU(H105A)	12.4	0.54

We hypothesized that the interaction between mtHSP70 and ISCU(N90A), which populates mainly the S-state, would be weaker than its interaction with ISCU. As anticipated, FMLR analysis of ^15^N TROSY-HSQC NMR spectra (Fig. 5, *D* and *E*) showed a much smaller effect: %S decreased from 95% in the absence of mtHSP70 to 83% at equimolar mtHSP70:U-^15^N-ISCU(N90A) (Fig. 5*F*). The magnitudes of the chemical shift perturbations upon adding mtHSP70 were smaller for U-^15^N-ISCU(N90A) than for U-^15^N-ISCU (Fig. 6*B*).

We also investigated the interaction between mtHSP70 and ISCU(H105A), a variant that favors the D-state. The %S of U-^15^N-ISCU(H105A) decreased from 12 to 0.54% upon the addition of equimolar mtHSP70 (Fig. 5, *G–I*; Table 2). The results again confirm that mtHSP70 preferentially binds to the D-state of ISCU.

##### HSC20 Preferentially Binds to the S-state of ISCU

**FIGURE 7.**
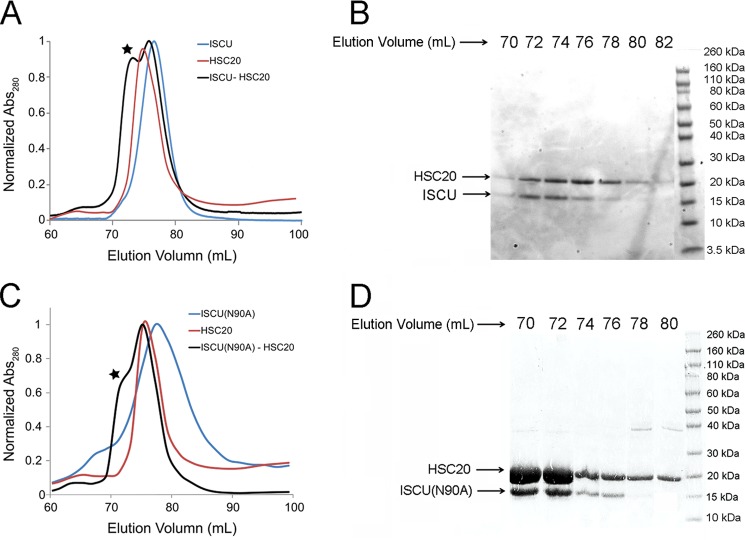
**Analytical gel filtration and SDS-PAGE show interaction between HSC20 and ISCU variants.** *A*, analytical gel-filtration elution profiles of ISCU alone (*blue line*), HSC20 alone (*red line*), and 2:1 mixture of HSC20:ISCU (*black line*). The peak at ∼70 ml elution volume (indicated by the *star*) is assigned to the HSC20-ISCU complex. *B*, SDS-PAGE of the gel-filtration elution fractions collected between 70 and 80 ml from the 2:1 HSC20:ISCU sample. The protein bands at ∼20 and ∼15 kDa correspond to HSC20 and ISCU, respectively. *C*, analytical gel-filtration elution profiles of ISCU(N90A) alone (*blue line*), HSC20 alone (*red line*), and 2:1 mixture of HSC20:ISCU(N90A) (*black line*). *D*, SDS-PAGE of the gel-filtration elution fractions collected between 70 and 82 ml from the 2:1 HSC20:ISCU(N90A) sample. The protein bands at ∼20 and ∼15 kDa correspond to HSC20 and ISCU(N90A), respectively.

**FIGURE 8.**
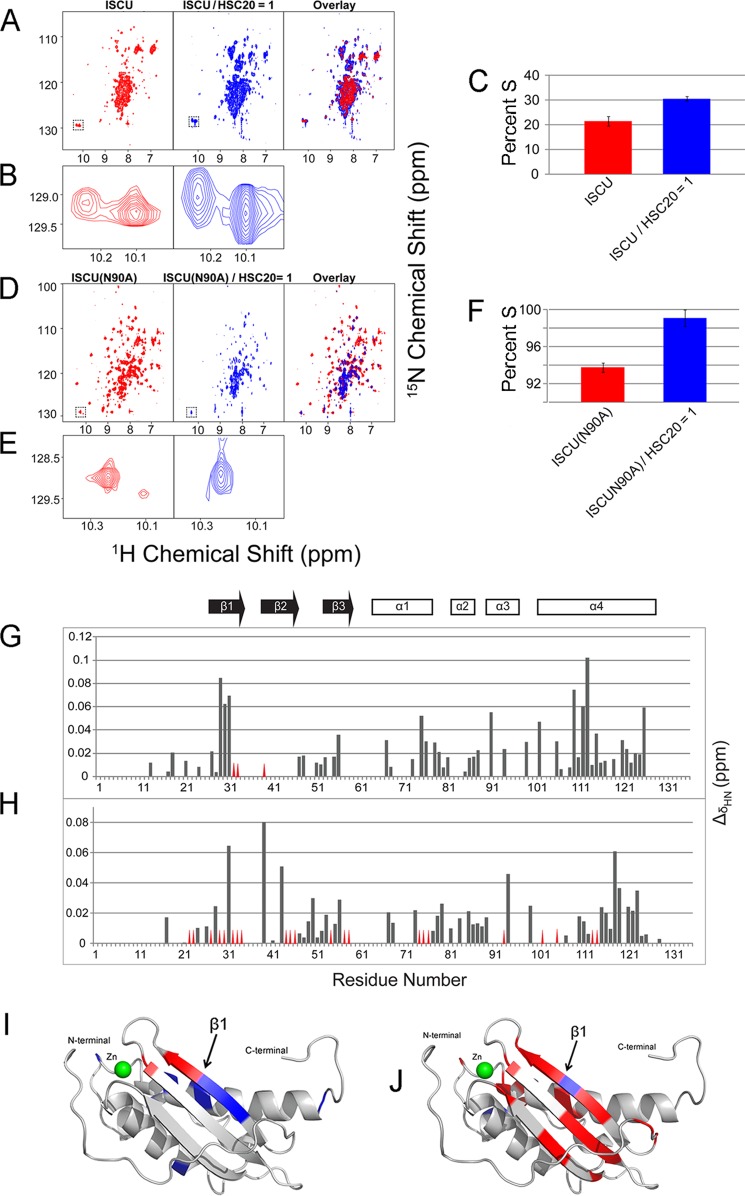
**Interaction between HSC20 and ISCU.** *A*, two-dimensional ^1^H,^15^N TROSY-HSQC spectra of U-^15^N-ISCU (*left panel*), U-^15^N-ISCU in the presence of equimolar unlabeled HSC20 and diluted by a factor of 2.25 (*middle panel*), overlay of the NMR spectra from the left and middle panels (*right panel*). *B*, expansions of the Trp-76 ^1^H,^15^N peaks from the spectra in *A. C*, %S calculated from FMLR analysis of the intensities of the Trp-76 cross-peaks assigned S and D under the conditions indicated. *D*, ^1^H,^15^N TROSY-HSQC spectra of U-^15^N-ISCU(N90A) (*left panel*), U-^15^N-ISCU(N90A) in the presence of 1 eq of unlabeled HSC20 and diluted by a factor of 2.25 (*middle panel*), overlay of the NMR spectra from the left panel and middle panels (*right panel*). *E*, expansions of the Trp-76 ^1^H,^15^N peaks from the spectra in *D. F*, %S calculated from FMLR analysis of the intensities of Trp-76 cross-peaks assigned S and D under the conditions indicated. *G*, chemical shift perturbation of ISCU signals (Δδ_HN_) upon the addition of 1.0 eq of HSC20 plotted as a function of ISCU residue number. *Red triangles* indicate residues whose NMR peaks were broadened beyond detection upon addition of HSC20. *H*, chemical shift perturbations (Δδ_HN_) for residues of ISCU(N90A) upon the addition of 1 eq of HSC20. *I*, chemical shift perturbations of ISCU signals resulting from HSC20 binding mapped onto the structure of Zn^2+^ bound *M. musculus* ISCU (PDB code 1WFZ) (47). Residues with Δδ_HN_ > 0.04 ppm are colored *blue*; residues whose NMR peaks were broadened beyond detection are colored *red. J*, chemical shift perturbations of ISCU(N90A) resulting from HSC20 binding mapped on the structure of Zn^2+^ bound *M. musculus* ISCU with color coding as in *I*.

Analytical gel-filtration chromatography was employed to investigate the interaction between ISCU and HSC20. Upon elution of a 2:1 (molar ratio) HSC20:ISCU mixture, a peak emerged at ∼70 ml. This elution volume corresponds to the expected molecular mass of the HSC20-ISCU complex (∼37 kDa) (Fig. 7*A*). SDS-PAGE of the elution fractions confirmed that the peak eluting at 70 ml contained both ISCU and HSC20 (Fig. 7*B*). We further followed two-dimensional ^15^N TROSY-HSQC NMR spectra of U-^15^N-ISCU upon titration with unlabeled HSC20 (Fig. 8, *A* and *B*). FMLR analysis showed that %S increased from ∼22 to ∼31% upon the addition of equimolar HSC20 (Fig. 8*C*). The results indicate that HSC20 binds preferentially to the S-state of ISCU, in analogy to the finding that *E. coli* HscB binds preferentially to the S-state of IscU (15). By transferring the backbone assignments for ISCU(N90A) to the ^1^H,^15^N HSQC spectrum from the S-state of ISCU, we were able to follow chemical shift perturbations Δ**δ**_HN_ (as given by Equation 2) of U-^15^N-ISCU upon titration with unlabeled HSC20 (Fig. 8, *G* and *I*),

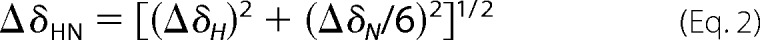
 where Δ**δ**_H_ and Δ**δ**_N_ are the chemical shift changes in the ^1^H and ^15^N dimensions, respectively.

**FIGURE 9.**
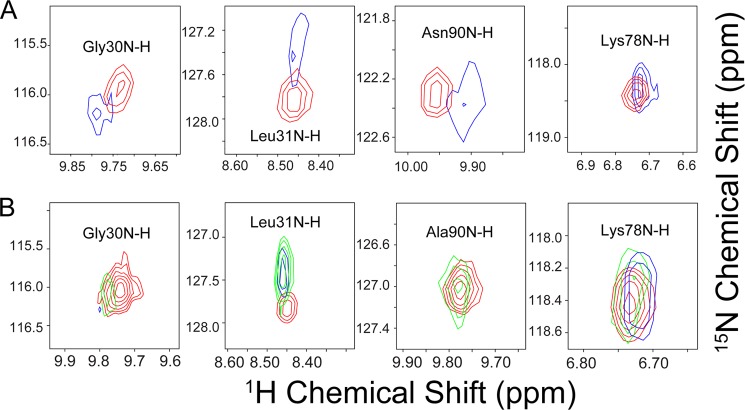
**Two-dimensional backbone ^1^H,^15^N NMR peaks from U-^15^N-ISCU variants corresponding to selected residues (Gly-30, Leu-31, Asn-90, and Lys-78, from *left* to *right*) that exhibit chemical shift changes upon binding HSC20.** *A*, U-^15^N-ISCU alone (*red*) and with 1.0 eq of unlabeled HSC20 (*blue*). *B*, U-^15^N-ISCU(N90A) alone (*red*) and with 0.5 eq (*green*) and 1.0 eq (*blue*) of unlabeled HSC20.

Examples of the several peaks from U-^15^N-ISCU that exhibited chemical shift perturbation upon binding HSC20 are shown in Fig. 9*A*. The residues of ISCU showing the largest chemical shift perturbations (Leu-31 and Val-32) (Fig. 8*G*) correspond to hydrophobic residues on the first β-strand of the three-dimensional structure of *M. musculus* Zn^2+^-ISCU (PDB code 1WFZ) (Fig. 8*I*). We speculate that the first β-strand of ISCU provides the binding interface for the ISCU-HSC20 interaction.

We also investigated the interaction between HSC20 and the S-state favoring ISCU variant ISCU(N90A). Similar to ISCU, analytical gel-filtration results showed the elution of a peak corresponding to the expected mass of the HSC20-ISCU(N90A) complex (∼37 kDa) (Fig. 7*C*). SDS-PAGE of the eluted fractions confirmed that this peak contained both ISCU and HSC20 (Fig. 7*D*). Two-dimensional ^15^N TROSY-HSQC NMR spectra of U-^15^N-ISCU(N90A) were acquired as a function of added unlabeled HSC20 (Fig. 8, *D* and *E*). Several peaks from U-^15^N-ISCU(N90A) exhibited chemical shift perturbations upon binding HSC20, and examples of these are shown in Fig. 9*B.* %S increased from ∼94 to ∼99% after the addition of 1 eq of HSC20, confirming that HSC20 binds preferentially to the S-state of ISCU(N90A) (Fig. 8*F*). Although more peaks of ISCU(N90A) were broadened beyond detection upon binding HSC20 as indicative of a tighter complex, the pattern of chemical shift perturbations (Fig. 8*H*) was similar to that for ISCU, suggesting that the first β-strand of ISCU(N90A) interacts with HSC20 (Fig. 8*J*).

##### In Vitro Fe-S Cluster Assembly Assay

**FIGURE 10.**
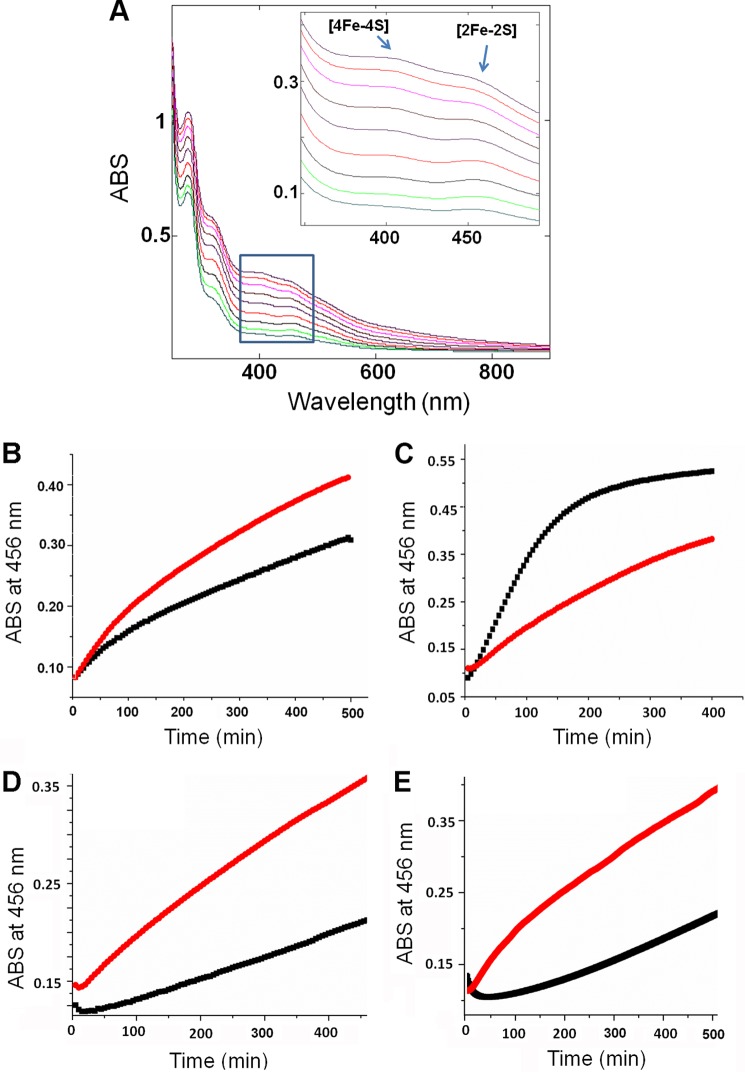
***In vitro* Fe-S cluster assembly assays.** *A*, UV-visible absorption spectra of ISCU during Fe-S cluster assembly catalyzed by NFS1-ISD11. Spectra were collected at 60-min intervals. Absorption (*ABS*) at 400 nm and 456 nm are characteristic of [4Fe-4S] and [2Fe-2S] clusters, respectively. *B*, time course of Fe-S cluster assembly followed at 456 nm. *Black line*, reaction containing ISCU and catalyzed by NFS1 alone. *Red line*, reaction containing ISCU and catalyzed by NFS1-ISD11. *C*, time course of Fe-S cluster assembly followed at 456 nm. *Black line*, reaction containing ISCU and a catalytic amount of *E. coli* IscS. *Red line*, reaction containing ISCU and a catalytic amount of NFS1-ISD11. *D*, time course of Fe-S cluster assembly followed at 456 nm. *Red line*, reaction containing ISCU and a catalytic amount of NFS1-ISD11. *Black line*, reaction containing ISCU(N90A) and a catalytic amount of NFS1-ISD11. *E*, time course of Fe-S cluster assembly followed at 456 nm in the presence of a catalytic amount of NFS1-ISD11. *Red line*, reaction containing ISCU. *Black line*, Reaction containing ISCU and 1 eq of Zn^2+^.

To investigate the physiological importance of these findings, we carried out *in vitro* Fe-S cluster assembly assays. The UV spectra of the assembly mixture collected as a function of time showed the growth of peaks at 456 and 400 nm, which are characteristic for [2Fe-2S] and [4Fe-4S] clusters, respectively (47). This result indicates that ISCU serves as the scaffold protein for both types of Fe-S cluster (Fig. 10*A*). We found that NFS1 alone could catalyze Fe-S cluster assembly on ISCU and that the addition of ISD11 increased the Fe-S cluster assembly rate (Fig. 10*B*). We also investigated *in vitro* Fe-S cluster assembly on ISCU catalyzed by *E. coli* cysteine desulfurase (IscS) and found that the assembly rate was faster than that catalyzed by an equivalent concentration of NFS1-ISD11 (Fig. 10*C*). To investigate the effect of the conformational states of ISCU on cluster assembly, we repeated the reaction replacing ISCU by ISCU(N90A), a variant that is primarily in the S-state. Compared with wild-type ISCU, cluster assembly on ISCU(N90A) occurred at a much slower rate (Fig. 10*D*). The addition of Zn^2+^, which stabilizes the S-state (data not shown), inhibited the rate of cluster assembly on ISCU (Fig. 10*E*).

##### ISCU and HSC20 Stimulate the ATPase Activity of mtHSP70

**FIGURE 11.**
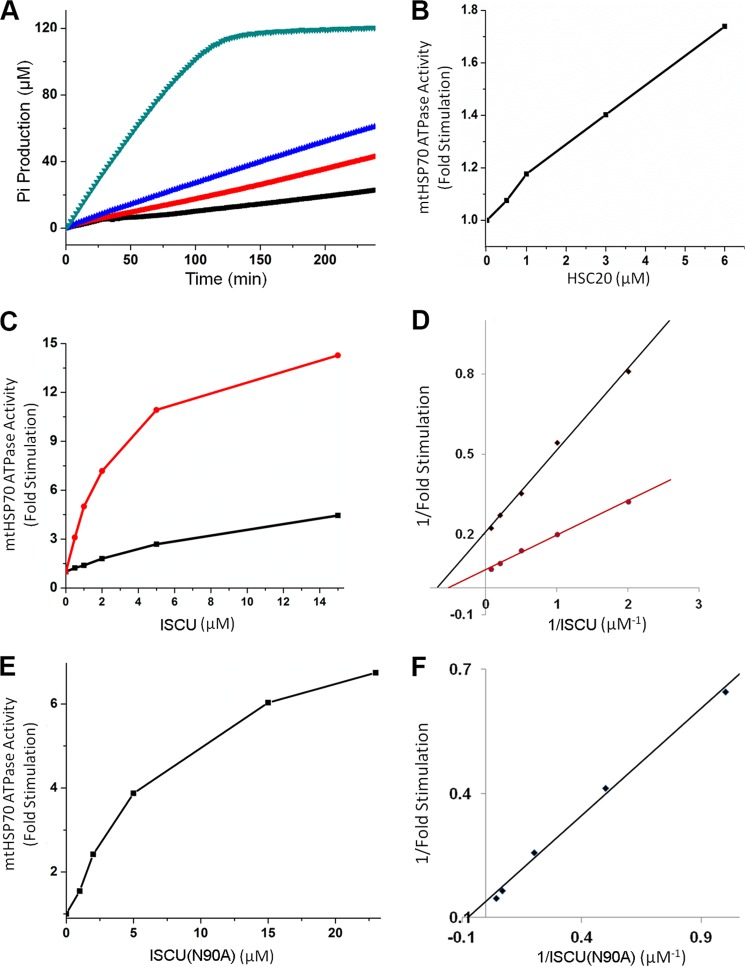
**Stimulation of the ATPase activity of mtHSP70 at 25 °C by ISCU and HSC20.** *A*, time course of ATP hydrolysis catalyzed by 1 μm mtHSP70 alone (*black line*) in the presence of 6 μm HSC20 (*red line*), in the presence of 15 μm ISCU (*blue line*), or in the presence of 4 μm HSC20 + 15 μm ISCU (*green line*). *B*, ATPase activity of 1 μm mtHSP70 as a function of added HSC20. *C*, ATPase activity of 1 μm mtHSP70 as a function of added ISCU in the absence of HSC20 (*black line*) and in the presence of 4 μm HSC20 (*red line*). *D*, double-reciprocal plot of the data of *C. E*, ATPase activity of 1 μm mtHSP70 as a function of added ISCU(N90A) in the presence of 5 μm HSC20. *F*, double-reciprocal plot of the data of Fig. 11*E.*

We found that mtHSP70 exhibited a basal ATPase activity of ∼0.10 ± 0.023 min^−1^ (Fig. 11*A*, *black*) which is lower than that reported for *E. coli* HscA (∼0.46 min^−1^) (49). The addition of either 6 μm HSC20 or 15 μm ISCU to 1 μm mtHSP70 increased the basal ATPase activity of mtHSP70 by factors of ∼1.7 and ∼4.5, respectively (Fig. 11*A, red and blue*). The addition of both 15 μm ISCU and 4 μm HSC20 increased the basal ATPase activity of mtHSP70 by a factor of ∼15 (Fig. 11*A, green*). Furthermore, we measured the effects of increasing concentrations of HSC20 alone (Fig. 11*B*), ISCU alone, and HSC20+ISCU (Fig. 11*C*) on the ATPase activity of 1 μm mtHSP70. Based on the double-reciprocal plot, ISCU alone elicited a maximal stimulation of ∼5 fold, and half-maximal stimulation occurred at ∼1.5 μm ISCU. In the presence of 5 μm HSC20, a maximal stimulation of ∼17 fold was observed upon adding ISCU, and the concentration of ISCU required for half-maximal stimulation was ∼2 μm (Fig. 11*D*). In the presence of 5 μm HSC20, the addition of 24 μm ISCU(N90A) increased the basal ATPase activity of mtHSP70 only by a factor of ∼7 (Fig. 11*E*). Based on the double-reciprocal plot, the maximal stimulation was ∼8 fold, and the concentration of ISCU(N90A) required for half-maximal stimulation was ∼3.5 μm (Fig. 11*F*).

## DISCUSSION

The best studied ISC system for Fe-S cluster biosynthesis is from bacteria. An NMR structure has been determined for the S-state of *E. coli* IscU (50), and x-ray and NMR structures have been determined for Zn^2+^ complexes of *Haemophilus influenzae* IscU, *Streptococcus pyogenes* IscU (51), and *Bacillus subtilis* IscU (PDB code 1XJS, 10.2210/pdb1xjs/pdb). X-ray structures have been determined for the *Aquifex aeolicus* IscU-[2Fe-2S] complex (17), for *E. coli* IscS (12), for the *E. coli* IscS-IscU complex (53), for *E. coli* HscB (54), for the substrate binding domain of *E. coli* HscA complexed with the IscU recognition peptide ELPPVKIHC (55), and for *E. coli* IscA (56). In addition, NMR studies of the *E. coli* ISC system have elucidated the roles of the S and D conformational states of IscU in the cycle of Fe-S cluster assembly and delivery (15, 16, 18, 50, 57). Despite sequence similarities of the homologous human mitochondrial proteins, the only three-dimensional structure determined to date of a protein from the human ISC system is that of HSC20 (human HscB) (58).

Results presented here show strong parallels between the conformational properties of the human (ISCU) and *E. coli* (IscU) scaffold proteins as well as their functional properties. Both human ISCU and *E. coli* IscU can adopt two very different folded conformations; one more structured (S-state) and one partially disordered (D-state) (Fig. 2*A*). Thus, the scaffold protein can be categorized as a metamorphic protein (59). In both cases, the S ⇄ D equilibrium is affected by single site amino acid substitutions at positions 39, 90, and 105 (16) (Fig. 2, *B* and *C*). Human ISCU is much less structured (∼28%S) than *E. coli* IscU (∼80%S) under similar buffer, pH, and temperature conditions (35). Another interesting difference is that the D39A substitution, which stabilizes the S-state of *E. coli* IscU (16, 50) and has been found to stabilize Fe-S clusters in *Azotobacter vinelandii* IscU and *Schizosaccharomyces pombe* Isu1 (60), was found to favor the D-state of human ISCU. Because we had earlier seen a parallel between the S-state fraction and cluster stability in *E. coli* IscU (16), this result appears at odds with an earlier study, which reported that human ISCU(D39A) supported cluster formation, whereas ISCU did not (61).

Prior studies of the human cysteine desulfurase have focused on the NFS1-ISD11 complex, because of difficulties in isolating the two subunits (26, 62, 63). In this study we successfully expressed and purified the two proteins separately. We show here that isolated ISD11 does not interact directly with ISCU (Fig. 4*C*). On the other hand, isolated NFS1 binds to the D-state of ISCU (Fig. 4*A*, *middle panel*) as does the NFS1-ISD11 complex (Fig. 4*A*, *bottom panel*).

Human ISCU contains the conserved ^99^LPPVK^103^ motif (Fig. 1*A*) found in *E. coli* IscU, which is recognized by the substrate binding domain of *E. coli* HscA (64). We found that human mtHSP70 interacts with the D-state of ISCU (Fig. 5, *A* and *B*). This result is parallel to the interaction between *E. coli* HscA and the D-state of IscU (18). The addition of 1 molar eq of mtHSP70 shifted the S ⇄ D equilibrium of ISCU completely to the D-state (Fig. 5, *A* and *B*). By contrast, variant ISCU(N90A), which has a stabilized S-state, remained primarily in the S-state upon the addition of 1 molar eq of mtHSP70 (Fig. 5, *C* and *D*).

Human HSC20, the putative human homolog of the specialized DnaJ type co-chaperones, has been reported to be involved in human mitochondrial Fe-S cluster biogenesis and mitochondrial iron homeostasis (34). Although human HSC20 and *E. coli* HscB share high structural similarity, the former contains an extra N-terminal rubredoxin-like domain not present in *E. coli* HscB or *S. cerevisiae* Jac1 (58). We found that human HSC20 preferentially binds and stabilizes the S-state of ISCU (Fig. 8, *A–F*). This result is parallel to the preferential interaction between *E. coli* HscB and the S-state of IscU (15). Residues on the first β-strand of ISCU, namely Gly-30, Leu-31, and Val-32, exhibited large chemical shift perturbations upon the addition of HSC20 (Fig. 8, *G–J*). We speculate that these residues are involved in a hydrophobic interaction between ISCU and HSC20.

The cluster assembly assay indicated that both [2Fe-2S] and [4Fe-4S] clusters could be assembled on ISCU (Fig. 10*A*) as catalyzed by NFS1 alone or by the NFS1-ISD11 complex (Fig. 10*B*). The rate with NFS1-ISD11 was ∼27% faster than with NFS1 alone. We found that *E. coli* IscS also assembled clusters on human ISCU at an even faster rate than with NFS-ISD11 (Fig. 10*C*). This result is in agreement with a recent finding that the human NFS1-ISD11 complex, in the absence of frataxin or its bacterial homologue CyaY, exhibited lower cysteine desulfurase activity than *E. coli* IscS (62). As catalyzed by NFS-ISD11, ISCU(N90A), the variant with a stabilized S-state, assembled clusters 2.5 times more slowly than ISCU (Fig. 10*D*). Similar results have been reported for *E. coli* IscU variants with stabilized S-states (16). As with the *E. coli* system (16), Zn^2+^ can also stabilize the S-state of human ISCU (data not shown), and the addition of Zn^2+^ was found to inhibit cluster formation (Fig. 10*E*).

The ATPase assay showed that mtHSP70 has a basal ATPase activity of ∼0.10 min^−1^ at 25 °C, which is close to that of *S. cerevisiae* Ssc1 and *E. coli* DnaK (∼0.12 min^−1^) (52) but much lower than that of *E. coli* HscA (0.46 min^−1^) (49). The lower ATPase activity of mtHSP70 compared with *E. coli* HscA can be attributed to the fact that mtHSP70 requires a nucleotide exchange factor (GrpEL1), which catalyzes the exchange of ADP for ATP, to reach maximal ATPase activity (48). The *E. coli* HscA/HscB chaperone system does not utilize a nucleotide exchange factor (14). ISCU and HSC20 individually enhanced the ATPase activity of mtHSP70 severalfold, and HSC20 plus ISCU together increased the ATPase activity still more (Fig. 11*A*). The synergic effect of HSC20 and ISCU in stimulating mtHSP70 ATPase activity is similar to that reported for stimulation of the ATPase activity of *E. coli* HscA by HscB and IscU (46). Unlike the *E. coli* system, in which the presence of HscB decreased the concentration of IscU required to stimulate the ATPase activity of HscA (46), the concentration of ISCU needed for half-maximal stimulation of mtHSP70 ATPase activity was the same or higher in the presence of HSC20 (Fig. 11, *C* and *D*). We found that ISCU(N90A) (95 %S) in the presence of HSC20 was half as effective as wild-type ISCU (28 %S) in stimulating the ATPase activity of mtHSP70 (Fig. 11, *E* and *F*), in agreement with our model in which the D-state of ISCU binds preferentially to mtHSP70. Together, these results are consistent with the proposed function of human mtHSP70 and HSC20 as the chaperone and co-chaperone, respectively, for human mitochondrial Fe-S cluster biosynthesis.

**FIGURE 12.**
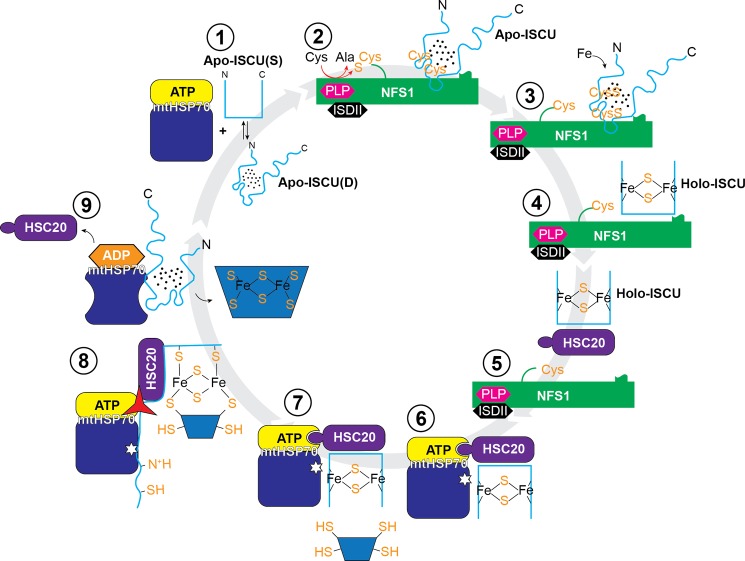
**Working model for human mitochondrial Fe-S cluster biogenesis.** *1*, apoISCU in D⇄S equilibrium. *2*, complex formed between the cysteine desulfurase complex (NFS1-ISD11) and the D-state of ISCU. *3*, sulfur delivered to Cys residues of ISCU. *4*, addition of iron to form a [2Fe-2S] cluster stabilizes the S-state of ISCU. *5*, the co-chaperone (HSC20) binds to holo-ISCU displacing the cysteine desulfurase complex. 6, the J-domain of HSC20 binds to the ATPase domain of the chaperone (mtHSP70), bringing holo-ISCU close to the chaperone. *7*, an acceptor protein containing free Cys -SH groups approaches. *8*, attack of cysteine residues from the acceptor protein liberates residues of ISCU that bind to the chaperone leading to activation of its ATPase activity. *9*, conversion of ATP to ADP leads to a conformational change in the substrate binding domain of the chaperone, which then binds preferentially to the D-state of ISCU releasing the holo-acceptor protein and HSC20. *1*, exchange of mtHSP70-bound ADP with ATP (which involves an exchange factor, not shown) leads to the release of ISCU, which resumes its equilibrium between the S- and D-states.

The findings above support a working model for human mitochondrial Fe-S cluster biogenesis (Fig. 12) that is analogous to one proposed for the *E. coli* system (35). In this model, conversion of ISCU to the D-state when bound to the cysteine desulfurase ensures that its Cys residues are free of metal (*e.g.* Zn^2+^) and capable of accepting sulfur. Cluster formation then stabilizes the S-state of ISCU, weakens its interaction with the cysteine desulfurase (NFS1-ISD11), and strengthens its interaction with the co-chaperone (HSC20), which binds preferentially to the S-state and targets the complex to the chaperone (mtHSP70). Then, the attack of an acceptor protein (*e.g.* apoferredoxin) triggers activation of the ATPase activity of the chaperone leading to conversion of ATP to ADP and a conformational change in the substrate binding domain of the chaperone to the form that binds the D-state of ISCU. The latter interaction ensures irreversible release of the cluster to the acceptor protein. Upon exchange of ADP for ATP (catalyzed by an exchange factor), ISCU is released to resume its S ⇄ D equilibrium.

## Footnotes

2 The abbreviations used are:

ISC: iron-sulfur cluster
ISCU: human scaffold protein
IscU: *E. coli* scaffold protein for Fe-S cluster biosynthesis
HSC20: human mitochondrial DnaJ-type co-chaperone
HscA: *E. coli* DnaK-type chaperone
HscB: *E. coli* DnaJ-type co-chaperone
HSQC: heteronuclear single quantum correlation
IscS: *E. coli* cysteine desulfurase
FMLR: fast maximum likelihood reconstruction
ISD11: small protein component of the human cysteine desulfurase complex
mtHSP70: human mitochondrial DnaK-type chaperone
NFS1: human mitochondrial cysteine desulfurase
TROSY: transverse relaxation optimized spectroscopy
IMAC: immobilized metal affinity chromatography.
